# Treatment outcomes of multidrug-resistant tuberculosis patients receiving ambulatory treatment in Shenzhen, China: a retrospective cohort study

**DOI:** 10.3389/fpubh.2023.1134938

**Published:** 2023-06-20

**Authors:** Ji Lecai, Peierdun Mijiti, Hong Chuangyue, Gao Qian, Tan Weiguo, Chen Jihong

**Affiliations:** ^1^Department of Tuberculosis Control, Shenzhen Center for Chronic Disease Control, Shenzhen, China; ^2^Department of Nephrology, Affiliated Bao'an Hospital of Shenzhen, The Second School of Clinical Medicine, Southern Medical University, Shenzhen, Guangdong, China; ^3^School of Public Health, Xinjiang Medical University, Urumqi, China; ^4^The Key Laboratory of Medical Molecular Virology of Ministries of Education and Health, School of Basic Medical Science, Fudan University, Shanghai, China; ^5^Department of Nephrology, The Second Affiliated Hospital of Shenzhen University, Shenzhen, China

**Keywords:** MDR-TB, ambulatory treatment, treatment outcomes, DOT, MDR/RR-TB

## Abstract

**Background:**

WHO recommended multidrug-resistant tuberculosis (MDR-TB) should be treated mainly under ambulatory model, but outcome of ambulatory treatment of MDR-TB in China was little known.

**Methods:**

The clinical data of 261 MDR-TB patients treated as outpatients in Shenzhen, China during 2010–2015 were collected and analyzed retrospectively.

**Results:**

Of 261 MDR-TB patients receiving ambulatory treatment, 71.1% (186/261) achieved treatment success (cured or completed treatment), 0.4% (1/261) died during treatment, 11.5% (30/261) had treatment failure or relapse, 8.0% (21/261) were lost to follow-up, and 8.8% (23/261) were transferred out. The culture conversion rate at 6 months was 85.0%. Although 91.6% (239/261) of patients experienced at least one adverse event (AE), only 2% of AEs caused permanent discontinuation of one or more drugs. Multivariate analysis showed that previous TB treatment, regimens containing capreomycin and resistance to FQs were associated with poor outcomes, while experiencing three or more AEs was associated with good outcomes.

**Conclusion:**

Good treatment success rates and early culture conversions were achieved with entirely ambulatory treatment of MDR-TB patients in Shenzhen, supporting WHO recommendations. Advantageous aspects of the local TB control program, including accessible and affordable second-line drugs, patient support, active monitoring and proper management of AEs and well-implemented DOT likely contributed to treatment success rates.

## Introduction

Multidrug-resistant or rifampicin-resistant TB (MDR/RR-TB), is an important obstacle to global TB control. The failure to diagnose and effectively treat MDR/RR-TB patients perpetuate ongoing MDR/RR-TB transmission in the community and can amplify the MDR/RR-TB burden (1). Although detection of MDR/RR-TB and treatment enrollment have improved globally over the past decades, the global treatment success rate (defined as cured or treatment complete) for MDR/RR-TB was still only 59% in 2020 (2).

The low global success rates may be partially attributed to the use of conventional MDR/RR-TB treatment regimens using second-line injectable drugs (SLIDs) that are lengthy – lasting 18–24 months, difficult to tolerate and associated with many adverse events (AE) (3). To try to improve treatment outcomes of MDR/RR-TB, recent efforts have focused on shortening treatment duration and using fully oral regimens that include novel and repurposed anti-TB drugs (bedaquiline, linezolid and clofazimine) (4–8). While these changes in the drug regimen and duration of treatment have greatly improved treatment outcome of MDR-TB patients, nearly 80% of success rates were also achieved with the long SLID-based regimens that were given as control arms in some clinical trials of these newer regimens (4, 9) and also in some programmatic studies (10, 11). The high success rates obtained in these studies with conventional long regimen was likely attributable to the high-quality care provided to patients, including free medications throughout the whole treatment course, individualized treatment based on drug susceptibility testing (DST), active monitoring and proper management of adverse events (AEs), and the availability of additional patient support (12, 13). This suggests the quality of care during MDR/RR-TB treatment plays an important role in improving treatment outcomes, irrespective of the composition and duration of the treatment regimen.

China has a large burden of MDR/RR-TB, accounting for 14% of MDR-TB cases globally in 2019. Although use of the new WHO recommended treatment regimens for MDR/RR is increasing in China, the treatment success rate in 2019 was still 54%, below the 59% global average (14). China has two models of care for treating MDR/RR-TB: a mixed model characterized by initial hospitalization for 1–2 months followed by ambulatory DOT at community health centers near the patient’s home; and a fully ambulatory model without hospitalization. The ambulatory, outpatient model is recommended by recent WHO treatment guidelines as cost-effective (14, 15), but treatment outcomes of the ambulatory model for MDR/RR-TB care in China have been poor, likely stemming from the poor quality of care during ambulatory treatment (16).

Shenzhen was one of the first cities supported by a Global Fund Project (GFP) for managing MDR-TB in China (2006–2014) (17). Most of the MDR-TB patients diagnosed in the municipal Shenzhen Center for Chronic Disease Control (CCDC) were treated in the fully outpatient model (18), and throughout the duration of the GFP there was an emphasis on providing high-quality ambulatory care. In this study, we retrospectively analyzed the clinical data of MDR-TB patients treated entirely as outpatients in Shenzhen during 2010–2015 in order to assess treatment outcomes and identify predictors of treatment success.

## Materials and methods

### Study design and settings

We performed a retrospective analysis of clinical data from MDR-TB patients enrolled on ambulatory treatment in the Shenzhen municipal CCDC between Jan 1st, 2010 and Dec 31th, 2015. Shenzhen is one of the most developed cities in China with a population of 20 million, nearly three quarters of whom are internal migrant workers. In Shenzhen, TB control is managed by one municipal, city-level CCDC, nine district-level CCDCs and one designated TB hospital. Drug resistant-TB (DR-TB) is diagnosed and treated by the municipal CCDC with the ambulatory, outpatient model (18).

### Study population and data collection

MDR-TB patients treated under the ambulatory model by the Shenzhen municipal CCDC during 2010–2015 period were included in this study. The eligibility criteria included: pulmonary TB with positive sputum cultures for *Mycobacterium tuberculosis* (MTB) and resistance to at least isoniazid (INH) and rifampin (RIF); aged 18 years or older; a negative test for human immunodeficiency virus (HIV) infection; availability of complete medical records; treatment under the ambulatory model; and signed informed consent to be treated within the GFP framework. The medical records of all eligible patients were reviewed, and clinical and demographic data were collected.

The study was approved by the Ethics Committee of the Shenzhen municipal CCDC, was performed with strict confidentiality of patient data and complied with the Helsinki statement. Due to the retrospective nature of the study, the Ethics Committee waived the requirement for patient consent to be included in this study.

### MDR-TB diagnosis and treatment

The management of DR-TB in Shenzhen during the study period followed the GFP guidelines. Diagnosis of TB in Shenzhen was usually made in the district-level CCDCs. As a routine practice, positive cultures from all district-level CCDCs were sent to the Shenzhen municipal CCDC reference laboratory for MTB identification and DST for INH and RIF by the proportional method in 2010–2011 and by the line probe assay in 2012–2015.

A team of pulmonary physicians at the municipal CCDC were responsible for diagnosing and treating MDR-TB, and patients diagnosed with MDR-TB were enrolled in treatment after giving informed consent. Phenotypic DST was routinely performed after enrollment in treatment, using the proportional method to determine susceptibility of MTB isolates to second-line drugs. MDR-TB treatment was individualized based on DST results and previous drug exposure. If DST to second-line drugs was not available at the initiation of treatment, an empiric treatment regimen was given based on previous exposure to second-line drugs and local epidemiology of second-line drug resistance. Patients were treated with 4–5 effective anti-TB drugs, including at least one FQ and one SLID, following the GFP guidelines (17). The duration of MDR-TB treatment was 24 months, with a 6-month intensive phase (8 months if sputum was positive at 6 months), followed by an 18-month continuation phase.

### DOT and monitoring of drug safety and response to treatment

Each patient was assigned a clinician from the municipal CCDC and a DOT provider from their community health-care center to ensure treatment adherence and promptly manage AEs associated with anti-TB drugs. Face-to-face DOT was strictly provided by a health professional in the community health centers at least 5 days a week. Patients visited the municipal CCDC clinic every month during the intensive phase, and every 2 months during the continuation phase for follow-up examinations and laboratory tests including: sputum culture; chest radiography (or chest computed tomography); routine urine tests; blood tests for hematology, electrolytes, liver, renal and thyroid function; audiometric and visual examination. In addition, during the daily DOT visits the patients were questioned by the community DOT provider about symptoms or signs suggestive of AEs.

### Additional patient support and care

Anti-TB drugs, follow-up examinations and laboratory tests were provided free to patients in Shenzhen during study period. At the initiation of treatment, TB patients and their families were given health education about TB disease, MDR-TB treatment, the importance of treatment adherence, TB transmission, coughing etiquette and proper disposal of sputum, by local CCDC staff and DOT providers. Additionally, education on AEs such as types of AEs, reporting procedure, and handling measures was given. Psychological support and counseling to strengthen self-esteem through empathy, trust, and encouragement, were provided to patients as needed.

### Variables and definitions

In this study, we collected the demographic and clinical data of all eligible patients, these included: age, gender, body mass index (BMI), treatment history, comorbidities, chest radiography result, site of TB focus, previous exposure to first and second-line TB drugs and smoking and drinking status. The definitions of AEs were described in detail elsewhere (19). Sputum culture conversion was defined as two consecutive negative cultures taken at least 30 days apart following an initial positive culture. Time to culture conversion was defined as the time interval between the date of MDR-TB treatment initiation and the date of the first of two consecutive cultures. Treatment outcomes were assessed at 30 months after the initiation of MDR-TB treatment. Treatment outcomes were categorized according to standard WHO definitions: cure, completed treatment, death during treatment, failure or relapse, loss to follow-up and transferred out (20). Cure or treatment completion were classified as treatment success, while death during treatment, treatment failure, relapse, loss to follow-up and not evaluated were classified as unfavorable outcomes.

## Results

### Characteristics of study participants

**Figure 1:**
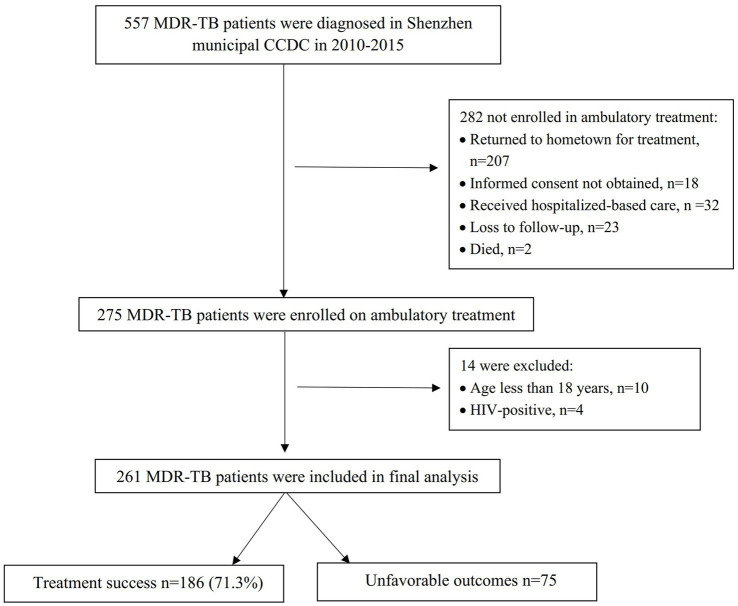
Flow diagram of study Population. MDR-TB, multidrug-resistant TB.

A total of 557 patients were diagnosed with MDR-TB in the Shenzhen municipal CCDC during 2010–2015, and of these, 275(49.4%) were enrolled in ambulatory treatment. The reasons for not enrolling in ambulatory treatment are listed in Figure 1. A comparison of the demographic characteristics between those enrolled in treatment and those not enrolled is shown in Supplementary Table 1. Of the patients enrolled in ambulatory treatment, 261 were included in the final analysis (Figure 1). The characteristics of the patients included in the analysis are shown in Table 1.

**Table 1:** Baseline characteristics of 261 MDR-TB cases in Shenzhen.

Characteristics	Numbers(n)	Proportion (%)
Gender		
Male	154	59.0
Female	107	41.0
Age group (years)		
≤30	135	51.7
31–40	83	31.8
≥41	43	16.5
Body Mass Index (kg/m^2^)		
<18.5	88	33.7
≥18.5	173	66.3
Smoking (Yes)	42	16.1
Alcohol use (Yes)	24	9.2
Diabetes (Yes)	9	3.5
Chronic hepatitis B or C (Yes)	29	11.2
Case classification		
New cases	71	27.9
Previously treated cases	183	72.1
Chest Radiography		
Bilateral involvement	157	60.1
Presence of cavity	126	48.3
Site of TB focus		
Pulmonary only	232	88.9
Pulmonary and extra-pulmonary	29	11.1
Previous exposure to FQs (≥1 month)	22	8.5
Previous exposure to SLIDs (≥1 month)	31	11.9
Drug resistance pattern		
Simple MDR-TB	164	69.8
Pre-XDR	67	28.5
XDR	4	1.7
missing	26	–
Use of regimen drugs		
Pyrazinamide	235	90.4
Levofloxacin	224	85.8
Moxifloxacin	28	10.7
Amikacin	176	67.4
Capreomycin	38	14.6
Ethambutol	166	63.6
Protionamide	230	88.1
Para-aminosalicylic acid	103	39.4
Amoxicillin/Clavulanate	6	2.3
Clarithromycin	13	5.0
Linezolid	7	2.7
Number of AEs per patients		
none	22	8.4
At least 1–2	138	52.9
3 and more	101	38.7

TB, tuberculosis; MDR-TB, multidrug-resistant TB; XDR-TB, extensive drug resistant TB; SLIDs, second-line injectable drugs; FQs, fluoroquinolones; AE, adverse events.

MDR-TB with resistance to any FQs and any SLIDs were classified as extensively drug resistant TB (XDR-TB). MDR-TB with resistance to only a FQ or only a SLID was classified as pre-XDR-TB.

### Pattern of anti-TB drugs resistance

The drugs used in the treatment regimens are shown in Table 1 and the results of DST to these drugs are shown in Table 2. A total of 231 (88.5%) patients had DST results for at least one FQ and 201 (77.0%) had DST results for at least one SLID. Of the 235 patients who had DST results for FQs or SLIDs, 24.1% were resistant to a FQ, 4.2% were resistance to a SLIDs, and 1.8% were resistant to both a FQ and a SLID and therefore XDR-TB.

**Table 2:** Results of drug susceptibility testing (*n* = 261).

Anti-TB drugs	Number of patients tested (*n*, %)	Number of resistant (n, %)
First-line drugs		
Isoniazid	261/261 (100.0)	261/261 (100.0)
Rifampicin	261/261 (100.0)	261/261 (100.0)
Ethambutol	232/261 (88.9)	74/232 (31.9)
Streptomycin	237/261 (90.8)	158/237 (66.7)
SLIDs 201/261(77.0)^#^		13/201 (6.5)
Amikacin	201/261 (77.0)	9/201 (4.5)
Capreomycin	201/261 (77.0)	10/201 (5.0)
FQs	231/261 (88.5)^*^	59/231 (25.5)
Ofloxacin	220/261 (84.3)	55/220 (25.0)
Levofloxacin	23/261 (8.8)	2/23 (8.7)
Moxifloxacin	17/261 (6.5)	1/17 (6.5)
Others		
Protionamide	197/261 (75.5)	46/197 (23.4)
Para-aminosalicylic acid	203/261 (77.8)	8/203 (3.9)

SLIDs, second-line injectable drugs; FQs, fluoroquinolones.

# At least one of amikacin and capreomycin was tested.

* At least one of ofloxacin, levofloxacin, moxifloxacin was tested.

### Sputum culture conversion and treatment outcome

A total of 238 (91.2%) patients had sputum culture conversion, with a median time of 90 days (IQR:61–146). Culture conversion rates at 2, 4, and 6 months after treatment initiation were 25.3, 70.7 and 85.0% (Table 3). The difference in the culture conversion rates at 6 months between treatment success and poor outcomes was statistically significant (89.8% vs. 69.0%, log-rank test: *χ*^2^ = 11.68, *p* < 0.001). Of 261 cases who were included in the analysis, 186 (72.3%) had good outcomes: 157 (60.1%) were cured and 29 (11.2%) completed treatment. A total of 75 (28.7%) patients had unfavorable outcomes: one (0.4%) died during treatment; 30 (11.5%) had treatment failure or relapse; 21 (8.0%) were lost to follow-up; and 23 (8.8%) were transferred out with unknown treatment outcomes (Table 4).

**Table 3:** Sputum culture negative conversion at 2, 4, and 6 months after initiation of treatment (*n*, %).

	Total (*n* = 261)	Treatment success (*n* = 186)	Poor outcome (*n* = 75)
At 2 months	65 (25.3)	45 (25.8)	17 (24.1)
At 4 months	179 (70.7)	139 (74.7)	40 (59.2)
At 6 months	212 (85.0)	167 (89.8)	45 (69.0)
Median days (IQR)	90 (61–146)	84 (60–127)	109 (63–255)

IQR, interquartile range.

**Table 4:** Treatment outcomes of 261 MDR-TB patients (*n*, %).

Treatment outcomes	Total (*n* = 261)	New cases (*n* = 74)	Previously treated cases (*n* = 187)
Treatment success	186 (71.3)	62 (83.8)	124(66.3)
Cured	157 (60.2)	53 (71.5)	104(55.6)
Treatment completed	29(11.1)	9 (12.2)	20(10.7)
Died	1(0.4)	1 (1.4)	0(0.0)
Failure or relapse	23 (11.5)	5 (6.8)	25(13.4)
Loss to follow-up	21 (8.1)	4 (5.4)	17(9.1)
Transferred out	23(8.8)	2 (2.7)	21(11.2)

MDR-TB, multidrug-resistant TB.

### Frequency of AEs during treatment

A total of 239 (91.6%) patients experienced at least one AEs, with a median of two AEs per patient, IQR:1–3). Most of AEs occurred within 6 months after initiation of treatment, with a median time of 108 days (IQR:39–187). The most common AEs were liver function abnormalities (70%), defined as serum transaminases greater than the normal upper limit, followed by gastrointestinal disorders (40.6%), renal function impairment (33.0%), and arthralgias (29.9%). Management of AEs is shown in Table 5. Overall, 24.9% of patients required a change of MDR-TB treatment due to AEs, including drug substitution or temporary suspension of the offending drugs, and 1.9% of patients required permanent discontinuation of treatment due to AEs. 16.7% of treatment failures were attributed to AEs.

**Table 5:** AEs occurred during treatment in total (*n*, %).

Type of AEs	Frequency of AEs	Management of AEs		
		Symptomatic treatment	Drug substitution, or temporary discontinuation of the offending drugs	Permanent discontinuation of treatment
Liver function abnormality	185/261 (70.9)	136/185 (73.5)	47/185 (25.4)	2/185 (1.1)
Gastrointestinal disorders	106/261 (40.6)	78/106 (73.6)	24/106 (22.6)	4/106 (3.8)
Renal function impairment	86/261 (33.0)	69/86 (80.2)	16/86 (18.6)	1/86 (1.2)
Arthralgia	77/261 (29.5)	56/77 (72.7)	21/77 (27.3)	0/77 (0.0)
Dermatologic disorders	33/261 (12.6)	25/33 (75.8)	8/33 (24.2)	0/33 (0.0)
Ototoxicity	28/261 (10.7)	8/28 (28.6)	16/28 (57.1)	4/28 (14.3)
Hematologic disorders	28/261 (10.7)	25/28 (89.3)	3/28 (10.7)	0/28 (0.0)
Visual impairment	16/261 (6.1)	11/16 (68.8)	4/16 (25.0)	1/16 (6.2)
Hypothyroidism	16/261 (6.1)	10/16 (62.5)	6/16 (37.5)	0/16 (0.0)
Peripheral neuritis	6/261 (2.3)	4/6 (66.7)	2/6 (33.3)	0/6 (0.0)
Psychiatric disorders	4/261 (1.5)	1/4(25.0)	3/4(75.0)	0/4(0.0)
Total number of AEs	585(100.0)	430/585 (73.5)	143/585 (24.4)	12/585 (2.1)
Abbreviations: adverse events, AE				

### Univariate and multivariate analysis of predictors of treatment success

The associations between patient characteristics and treatment success are shown in Table 6. Multivariate analysis found that prior TB history (previously treated cases vs. new cases, OR = 0.42, 95% CI:0.20–0.86), regimens containing capreomycin (yes vs. no, OR = 0.49, 95% CI:0.24–0.99), and resistance to any FQ (yes vs. no, OR = 0.39, 95% CI:0.19–0.78) were all associated with poor outcomes. Interestingly, experiencing three or more AEs (3 or more vs. no AEs, OR = 3.78, 95% CI = 1.2–11.9) was associated with treatment success (Table 6).

**Table 6:** Univariate and multivariate analysis of predictors of treatment success (*n*, %).

Predictors	Treatment outcomes		Univariate^#^		Multivariate^#^	
	Treatment success (*n* = 186)	Poor outcome (*n* = 75)	ORs	95% CI	aORs	95% CI
Gender						
Male	102 (66.2)	52 (33.7)	1.00			
Female	84 (78.5)	23 (21.5)	1.86	1.05–3.29		
Age group (years)						
≤30	98 (72.6)	37 (27.4)	1.00			
31–40	59 (71.1)	24 (28.9)	0.93	0.51–1.70		
≥41	29 (67.4)	14 (32.6)	0.78	0.37–1.64		
Body mass index (kg/m^2^)						
<18.5	60 (68.2)	28 (31.8)	1.00			
≥18.5	126 (72.8)	47 (27.3)	1.25	0.71–2.19		
Smoking						
Yes	27 (64.3)	15 (35.7)	0.67	0.33–1.36		
No	159 (72.6)	60 (27.4)	1.00			
Alcohol use						
Yes	14 (58.3)	10 (41.7)	0.52	0.22–1.25		
No	172 (72.6)	65 (27.4)	1.00			
Diabetes						
Yes	5 (55.6)	4 (44.4)	0.49	0.13–1.87		
No	181 (71.8)	71 (28.2)	1.00			
Chronic hepatitis B or C						
Yes	13 (59.1)	9 (40.9)	0.55	0.22–1.05		
No	173 (72.4)	66 (27.6)	1.00			
Case classification						
New cases	62 (83.8)	12 (16.2)	1.00		1.00	
Previously treated cases	124 (66.3)	63 (33.7)	0.38	0.19–0.76	0.42	0.20–0.86
Chest radiography						
Bilateral involvement (Yes vs. No)	107 (67.7)	51 (32.3)	0.64	0.36–1.12		
Presence of cavity (Yes vs. No)	87 (68.0)	41 (32.0)	0.72	0.42–1.25		
Use of Capreomycin						
Yes	22 (57.9)	16 (42.1)	0.49	0.24–0.99	0.44	0.20–0.99
No	164 (73.5)	59 (26.5)	1.00		1.00	
Resistant to any SLIDs (*n* = 201)						
No	143 (74.5)	49 (28.2)	1.00			
Yes	8 (61.5)	5 (38.5)	0.61	0.16–2.06		
Missing	39 (65.0)	21 (35.0)	0.57	0.35–1.08		
Resistant to any FQs (*n* = 231)						
No	127 (73.8)	45 (26.1)	1.00		1.00	
Yes	38 (64.4)	21 (35.6)	0.51	0.28–0.93	0.39	0.19–0.78
Missing	21 (70.0)	9 (30.0)	0.67	0.31–1.46	0.35	0.12–1.23
Frequency of AEs						
None	14 (63.6)	8 (36.4)	1.00		1.00	
At least 1–2	91 (65.9)	47 (34.1)	1.10	0.85–1.67	1.28	0.45–3.62
3 and more	81 (80.2)	20 (19.8)	2.31	0.85–6.27	3.78	1.20–11.93

TB, tuberculosis; MDR-TB; SLIDs, second-line injectable drugs; FQs, fluoroquinolones; AE, adverse events; OR, odds ratio; aOR, adjusted odds ratio.

# Univariate and multivariate logistic regression analysis were performed, only the variables significantly associated with treatment success on univariate analysis were put into a multivariate model. *p* < 0.20 was applied as threshold value of backward elimination.

## Discussion

During the duration of the GFP in Shenzhen from 2010 to 2015, the success rate of treating MDR-TB patients with ambulatory, conventional, SLID containing long regimens was 71.3%. This was significantly higher than the 52% global average during 2010–2015, and the 48.4% success rate in other GFP-supported regions of China (17). One major contributor to poor outcome of ambulatory treatment of MDR-TB in China was loss to follow-up (16). The percentage of MDR/RR-TB patients in our study who were lost to follow-up was 8%, which was lower than the 23% found in an analysis of observational studies of SLID-based, long regimens (21), and lower than with long SLID-based regimens in other regions of China (11.3–27%) (22, 23). Previous studies identified financial difficulties, limited knowledge of the disease, negative beliefs and attitudes about treatment, and lack of access to second-line drugs as factors associated with loss to follow-up among MDR/RR-TB patients (24). In Shenzhen however, a sustained supply of second-line drugs was guaranteed by GFP and all anti-TB drugs, laboratory tests and medical examinations were provided to patients without cost. In addition, MDR/RR-TB patients in our study were treated under an ambulatory model, so there were no costs related to hospitalization. These measures reduced the economic burden faced by patients and could have contributed to the low percentage of patients lost to follow-up. In addition, the GFP included patient education on treatment adherence, psychological support, and careful implementation of DOT, which also may have contributed to the relatively low, if still suboptimal, percentage of patients lost to follow-up (25).

The recently WHO-recommended all-oral shorter or longer regimens containing novel second-line drugs, might help to further reduce the number of patients lost to follow-up and improve treatment outcome. But currently these drugs are expensive in China and the reimbursement rate to the patient is usually low, and the access to the drugs is limited. The costs of WHO-recommended all-oral regimen containing bedaquiline, linezolid and clofazimine of 20 months could reach 300,00 US dollars in China, which is five times higher than the costs of conventional SLID-based regimen in our study (26). A study conducted in four developed regions of China found that bedaquiline and delamanid were available in only 50.0 and 2%, respectively, of MDR-TB designated hospitals, and stock-outs of other second-line anti-TB drugs was common (27). In less developed regions of China with high burdens of MDR/RR-TB, the accessibility and affordability of second-line drugs could be even worse (28). Overall, our findings suggest that sustained availability of affordable second-line drugs can be important contributors to MDR/RR-TB treatment adherence and treatment success in an ambulatory model and should be emphasized in the roll-out of WHO-recommended new regimens in China.

AEs are common during MDR-TB treatment, often leading to low quality of life and increasing the risk of treatment failure or loss to follow-up (29). In our study, although 91.6% of patients experienced at least one AE, similar to the findings in another study in China (19), most of AEs were cured or improved by symptomatic treatment and change of MDR-TB regimen, and very few patients (1.9%) required permanent discontinuation of treatment. In addition, experiencing three or more AEs is a predictor of treatment success in our study. We believe this might be attributable to patient education on AEs, and active monitoring and management of AEs in timely manner (30). Our study suggests that AEs occurring during MDR/RR-TB treatment can be adequately managed under the ambulatory model if there is active monitoring and prompt, proper attention to drug toxicities (31).

Multivariate regression also found that previous TB treatment or having a strain resistant to any FQ were associated with poor outcomes, consistent with other studies (32). In addition, the use of capreomycin was less likely to be associated with treatment success in our study. Recent WHO guidelines no longer recommend kanamycin and capreomycin for MDR/RR-TB treatment because they have been associated with poor outcomes (8, 33). In China, however, capreomycin is still recommended in DR-TB treatment guidelines (2019), partially due to its lower incidence of AEs compared to Amikacin (34). Future studies with larger sample sizes are needed to assess the effectiveness of capreomycin for treating MDR-TB in China.

This study had several limitations. Only half of MDR-TB patients diagnosed in Shenzhen were enrolled in treatment, which may have introduced a selection bias. Those enrolled in ambulatory treatment in Shenzhen were less likely to be new cases than those not in ambulatory treatment, whereas age, gender, and drug resistance patterns were similar (Supplementary Table S1). As treatment success rates were higher in new cases, this may have led to an underestimation of success rates achievable with ambulatory care. In addition, 11.3% of MDR-TB patients diagnosed by the Shenzhen municipal CDCC were transferred to other TB-designated Hospitals for hospital-based management. These were likely patients with severe MDR-TB or comorbidities associated with poor treatment outcomes, which would have led to an overestimation of the potential success of ambulatory treatment for the overall population of TB patients.

In conclusion, good success rates and early culture conversion were achieved in MDR-TB patients with SLID-based conventional long regimens under a fully ambulatory model during the GFP in Shenzhen. These findings support WHO recommendations that MDR/RR-TB patients should be treated as outpatients. However, we believe that the high success rates with the ambulatory based, conventional regimens in Shenzhen were likely attributable to the high quality of care that integrated sustained access to affordable drugs, well-implemented DOT, ample patient support and active monitoring and proper management of AEs. These aspects should be emphasized when implementing the newer, WHO recommended, all-oral MDR-TB treatment regimens.

## Data availability statement

The original contributions presented in the study are included in the article/Supplementary material, further inquiries can be directed to the corresponding author.

## Ethics statement

The studies involving human participants were reviewed and approved by Ethics Committee of the Shenzhen municipal CCDC. Written informed consent for participation was not required for this study in accordance with the national legislation and the institutional requirements.

## Author contributions

All authors listed have made a substantial, direct, and intellectual contribution to the work and approved it for publication.

## Funding

This study was funded by the Sanming project of Medicine in Shenzhen (SZSM201611030) and Guangdong Medical Science and Technology Research Fund (B2021075).

## Conflict of interest

The authors declare that the research was conducted in the absence of any commercial or financial relationships that could be construed as a potential conflict of interest.

## Publisher’s note

All claims expressed in this article are solely those of the authors and do not necessarily represent those of their affiliated organizations, or those of the publisher, the editors and the reviewers. Any product that may be evaluated in this article, or claim that may be made by its manufacturer, is not guaranteed or endorsed by the publisher.
