# Supplementary material for: Treatment outcomes of multidrug-resistant tuberculosis patients receiving ambulatory treatment in Shenzhen, China: a retrospective cohort study
Source: Front Public Health. 2023 Jun 20;11:1134938. doi: 10.3389/fpubh.2023.1134938 (PMC10319049; doi:10.3389/fpubh.2023.1134938)
Supplement: Supplementary file 1 [file Table_1.docx]

Supplementary Table 1. Comparison of characteristics between enrolled in ambulatory treatment and not enrolled in ambulatory treatment in Shenzhen municipal CDCC, 2010-2015 (n, %)

| Characteristics | Treatment enrollment | | χ^2^ | *P* |
| --- | --- | --- | --- | --- |
|  | Yes (n=261) | No (n=282) |  |  |
| Gender |  |  |  |  |
| Male | 154(59.0) | 171(60.6) | 0.15 | 0.70 |
| Female | 107(41.0) | 111(39.4) |  |  |
| Age group (years) | |  |  |  |
| ≤30 | 135(51.7) | 146 (51.8) | 5.04 | 0.08 |
| 31-40 | 83(31.8) | 71 (25.2) |  |  |
| ≥41 | 43(16.5) | 65 (23.0) |  |  |
| Case classification | |  |  |  |
| New cases | 71(27.9) | 136 (48.2) | 23.17 | <0.001 |
| Previously treated cases | 183(72.1) | 146 (51.8) |  |  |
| Drug resistance pattern |  |  |  |  |
| simple MDR-TB | 164(69.8) | 40 (61.6) | 2.85 | 0.24 |
| pre-XDR | 67(28.5) | 22 (33.8) |  |  |
| XDR | 4(1.7) | 3(4.6) |  |  |
| missing | 26 | 217 |  |  |
